# Evaluation and verification of the QFix Encompass^TM^ couch insert for intracranial stereotactic radiosurgery

**DOI:** 10.1002/acm2.12387

**Published:** 2018-06-15

**Authors:** Karen Chin Snyder, Ilma Xhaferllari, Yimei Huang, M. Salim Siddiqui, Indrin J. Chetty, Ning Wen

**Affiliations:** ^1^ Department of Radiation Oncology Henry Ford Health System Detroit MI USA

**Keywords:** couch model, dose calculation, immobilization, intracranial SRS, treatment planning

## Abstract

The QFix Encompass^TM^ stereotactic radiosurgery (SRS) immobilization system consists of a thermoplastic mask that attaches to the couch insert to immobilize patients treated with intracranial SRS. This study evaluates the dosimetric impact and verifies a vendor provided treatment planning system (TPS) model in the Eclipse TPS. A thermoplastic mask was constructed for a Lucy 3D phantom, and was scanned with and without the Encompass^TM^ system. Attenuation measurements were performed in the Lucy phantom with and without the insert using a pinpoint ion chamber for energies of 6xFFF, 10xFFF and 6X, with three field sizes (2 × 2, 4 × 4, and 6 × 6 cm^2^). The measurements were compared to two sets of calculations. The first set utilized the vendor provided Encompass TPS model (Encompass_TPS_), which consists of two structures: the Encompass and Encompass base structure. Three HU values for the Encompass (200, 300, 400) and Encompass Base (−600, −500, −400) structures were evaluated. The second set of calculations consists of the Encompass insert included in the external body contour (Encompass_EXT_) for dose calculation. The average measured percent attenuation in the posterior region of the insert ranged from 3.4%–3.8% for the 6xFFF beam, 2.9%–3.4% for the 10xFFF, and 3.3%–3.6% for the 6X beam. The maximum attenuation occurred at the region where the mask attaches to the insert, where attenuation up to 17% was measured for a 6xFFF beam. The difference between measured and calculated attenuation with either the Encompass_EXT_ or Encompass_TPS_ approach was within 0.5%. HU values in the Encompass_TPS_ model that provided the best agreement with measurement was 400 for the Encompass structure and −400 for the Encompass base structure. Significant attenuation was observed at the area where the mask attaches to the insert. Larger differences can be observed when using few static beams compared to rotational treatment techniques.

## 1 INTRODUCTION

Intracranial stereotactic radiosurgery (SRS) is a treatment technique used to deliver large doses of radiation to small targets in the cranium in order to manage primary brain tumors, metastasis, or functional diseases. Frameless mask‐based systems have become popular over the past decade since they are noninvasive; allowing for greater patient comfort as well as the ability to fractionate treatments while still retaining the immobilization accuracy of frame‐based treatments.^1^, ^2^, ^3^ Current frameless‐based systems typically use a clam shell style mask to immobilize the patient in order to provide submillimeter accuracy treatments to small intracranial lesions.^4^

Frameless systems use either extensions in which the mask system extends off the patient support structure, or overlays in which the mask system is attached and indexed to the carbon fiber patient support structure. The QFix Encompass^TM^ SRS immobilization system, created by QFix (Avondale, PA, USA) consists of a couch insert, and a thermoplastic mask attached to the raised component of the insert. The geometry and design of the insert is unique in that high density carbon fiber material surrounds the cranium, which may interfere with the target area to be treated.

Several groups have demonstrated the importance of modeling immobilization devices in the treatment planning system (TPS) to limit their dosimetric impact, particularly on skin dose, dose distribution, and attenuation.^5^, ^6^, ^7^, ^8^, ^9^, ^10^, ^11^ A TPS model of the QFix Encompass insert has been created and is available in the Eclipse TPS software, v15.5 (Varian Medical System, Palo Alto, CA, USA). The QFix Encompass immobilization device is an integral part of the Varian HyperArc^TM^ High‐definition radiotherapy automated SRS delivery workflow. The immobilization device allows the patient to be located in space relative to the machine isocenter to ensure machine clearance and efficiency during automated delivery. In this study, we evaluate the dosimetric properties of the QFix Encompass^TM^ system and quantify the amount of attenuation through the system. The Hounsfield Unit (HU) values of the couch model in the TPS were verified. Finally, we evaluated the dosimetric consequences and robustness of the system.

## 2 MATERIALS AND METHODS

### 2.A The QFix Encompass^TM^ model

**Figure 1:**
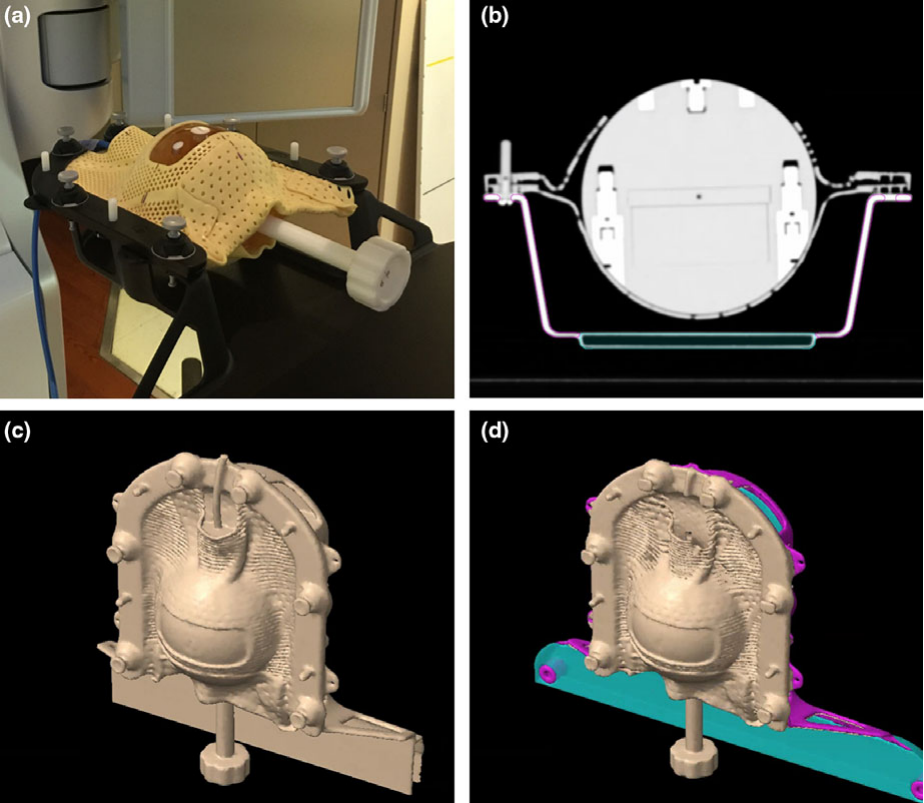
(a) Encompass insert and customized two pieces, clam shell style mask made for a Standard Imaging Lucy Phantom. (b) Axial cross‐section of the Lucy phantom in mask demonstrating the two portions of the model: Encompass (magenta) and the Encompass Base (cyan). (c) Encompass_EXT_ image set consisting of the Encompass insert included in the external contour for dose calculation. (d) Encompass_TPS_ image set consisting of the phantom and mask contoured in the external contour and the Encompass treatment planning structure model.

The QFix Encompass^TM^ SRS immobilization system consists of two parts: the Encompass insert and the clam shell style Fiberplast^TM^ mask [Fig. 1(a)]. The Encompass insert is an immobilization device that can be attached to or overlaid on the treatment couch. The clam shell style mask consists of an anterior and posterior portion which is customized for each patient during simulation. The Fiberplast^TM^ mask is a low temperature thermoplastic that hardens quickly, typically within 10 min. The mask is aligned to the insert with acrylic pins and locked into place with adjustable shims.

To evaluate the CT numbers of the Encompass system, a mask was made on the Lucy 3D QA phantom (Standard Imaging, Middleton WI, USA). After the mask hardened, a CT scan was acquired of the mask and Encompass insert using a Philips Brilliance Big bore scanner (Philips, Netherlands), using our institution's intracranial SRS protocol. (120 kVp, 400 mAs, 1 mm slice thickness, FOV = 350 mm, 512 × 512 Matrix). The HU values were evaluated for all portions of the Encompass system.

The QFix Encompass^TM^ insert is modeled in Eclipse TPS v15.5 as a support structure. The Encompass TPS model is a simplified model of the full Encompass system that does not include some portions of the couch. The Encompass TPS model consists of two separate structures: Encompass and Encompass Base. The “Encompass” structure includes the bulk portion of the carbon fiber U‐shaped insert. The “Encompass Base” structure includes the posterior region of the insert system and is made up of a double‐layered section with a hollow interior [Fig. 1(b)].

### 2.B Phantom setup

Measurements were performed in a spherical, Lucy 3D QA phantom (Standard Imaging, Middleton WI, USA) with a PTW Pinpoint ion chamber (Freiburg, Germany), 0.015 cc active volume. The Lucy phantom was immobilized by creating a custom mask in the Encompass insert system [Fig. 1(a)]. A CT scan of the phantom and Encompass insert was acquired using the SRS protocol. The scan was imported into the TPS, where two image sets were generated based on how the Encompass system was to be included for dose calculation.

The first image set was the Encompass TPS image set (Encompass_TPS_) which was contoured according to the vendor recommendations for incorporating the Encompass couch structure onto a patient image set. This consists of contouring the entire Encompass system in the external body contour, including the patient and mask. The Encompass TPS model is inserted as a support structure, and then removed from the external body contour using the Boolean tool. The contouring procedure results in the external body contour encompassing the patient and mask, and the Encompass insert as a separate support structure [Fig. 1(d)]. This allows the dose calculation to take into account portions of the mask system that are custom to each patient and are not included in the couch structure.

The second image set was the Encompass external image set (Encompass_EXT_) which was contoured according to our institution's policies and procedures, prior to availability of a couch model in the TPS. The external body contour includes all portions of the Encompass immobilization device [Fig. 1(c)]. The external was contoured using the search body function in Eclipse (Lower Threshold: −700 HU, Fill all cavities (2‐D All), Disconnect Radius [cm]: 0.50, Fill all Cavities, Close openings Radius [cm]: 2.00, Smoothing Level: 1). The final Encompass_EXT_ image set included the Lucy phantom, mask, and Encompass insert inside the external structure [Fig. 1(c)].

A third image set, Lucy_only_, was generated from a CT scan of the Lucy phantom by itself without the Encompass insert and imported directly into the TPS. Measurements in Lucy phantom with and without the Encompass insert were performed in various areas of the Encompass insert to quantify the amount of attenuation, which was then compared with calculated values from the three image sets above.

### 2.C Attenuation measurements

Measurements were performed on a Varian EDGE linear accelerator (Varian, Palo Alto, CA, USA) using three energies: a flattened 6 MV beam (6X), and flattening filter free 6 MV (6xFFF) and 10 MV (10xFFF) beams. The measurements were performed for each energy, with three field sizes: 2 × 2, 4 × 4, and 6 × 6 cm^2^. A total of 18 measurement setups were performed with and without the Encompass insert. The Encompass and Lucy setups were aligned using CBCT, matched to the TPS CT with 6 degrees of freedom.

**Figure 2:**
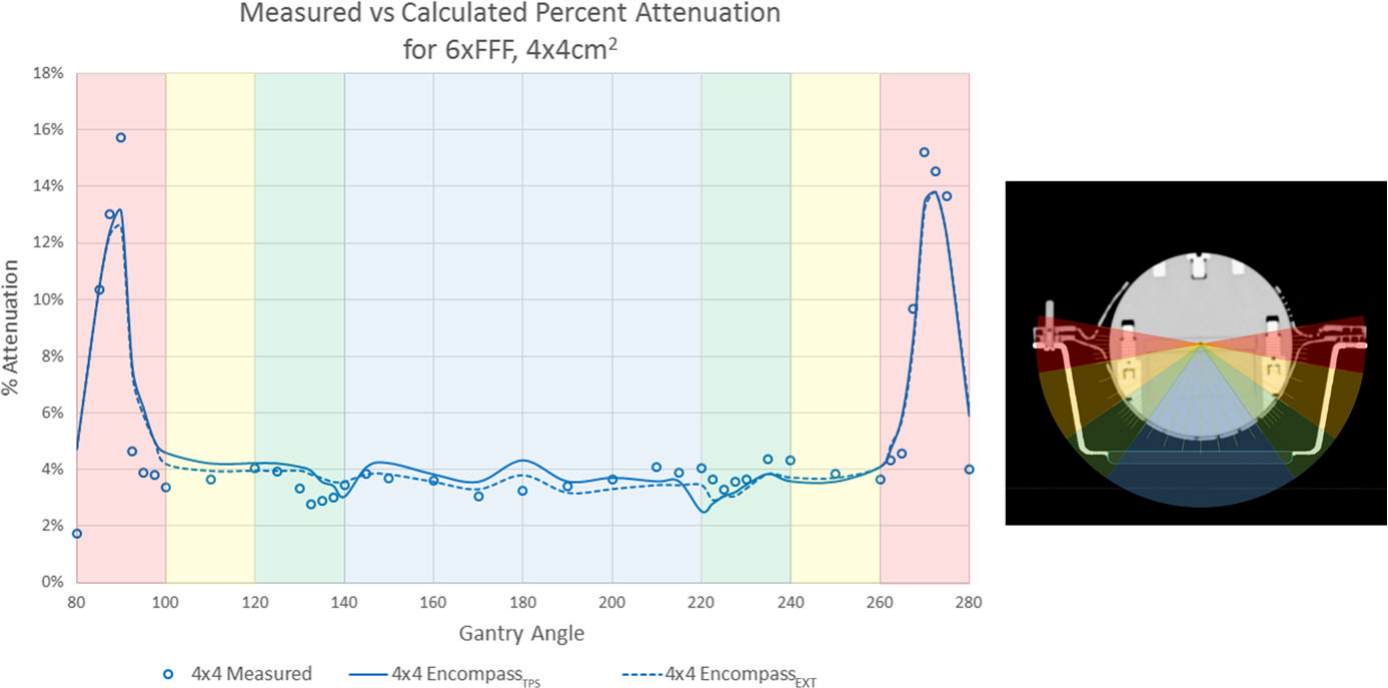
(Right) Axial cross‐section of Lucy phantom demonstrating measurement zones used for HU validation. Zone 1 (blue), Zone 2 (green), Zone 3 (yellow), and Zone 4 (red). (Left) Measured and calculated percent attenuation for Encompass_TPS_ and Encompass_EXT_ structure sets with Zones 1–4 highlighted to demonstrate areas of high attenuation.

A total of 41 measurements per energy and field size were performed. The measurements were broken down into four zones (Fig. 2). Zone 1 (blue) is a 70° area that represents the area of the “Encompass Base” structure in the Eclipse Encompass TPS model. Zone 1 ranges from gantry 140° to 220°, with measurements taken at increments of 5 or 10°. Zone 2 (green) is a 15° area and represents the transition zone between the Encompass and Encompass base structures. Zone 2 ranges from 125–140° to 220–235°, with measurements taken in increments of 2.5°. Zone 3 (yellow) is a region of 20° and represents the solid portion of Encompass insert. Zone 3 ranges from 100–120° to 240–260°, with measurements taken at increments of 10°. Zone 4 (red) spans a region of 12.5° in the area where the mask attaches to the Encompass insert. Zone 4 ranges from 262.5–275° to 85–97.5°, with measurements taken at 2.5° increments.

The measured percent attenuation, Atten_measured_, of the insert was calculated as (1−(Dose_Encompass_/Dose_Lucy_))*100. Where Dose_Encompass_ is the dose measured in Lucy with the Encompass insert and Dose_Lucy_ is the dose measured in the Lucy, without the Encompass insert, under the same irradiation conditions.

### 2.D Validation of HU Values for the Encompass TPS Model

The HU values for the Encompass TPS model were determined for each of the structures in the Encompass model by choosing the HU that minimized the difference between the measured and calculated percent attenuation in a specific zone. The HU value for the “Encompass Base” structure was determined from measurements in Zone 1, the region of double‐layered carbon fiber. The measurements were averaged and compared to the structure set HU values of −600, −500, and −400. Similarly, the HU value for the “Encompass” structure was determined by choosing the HU that minimized the difference between measured and calculated attenuation in Zone 3, the region that consists of only the carbon fiber insert. HU values of 200, 300, and 400 were evaluated in this study.

The calculation of the percent attenuation was performed in the Eclipse TPS v15.5 on the three image sets (Encompass_TPS_, Encompass_EXT_, and Lucy_only_) for three energies and three field sizes using the Anisotropic Analytical Algorithm v.15.5.02 with a grid size of 1.5 mm. The percent attenuation of the Encompass TPS model, Atten_Calc,_ was calculated as (1−(Dose_Encompass_/Dose_Lucy_))*100. Where Dose_Encompass_ is the dose calculated to the ion chamber in the Encompass_TPS_ image set and Dose_Lucy_ is the dose calculated to the ion chamber in the Lucy_only_ image set under same calculation conditions.

To verify that all portions of the Encompass system were correctly modeled in the Encompass TPS model, the percent attenuation was also calculated with the Encompass_TPS_ and compared to the percent attenuation calculated with the Encompass_EXT_ image set.

### 2.E Clinical case recalculation and measurement

Ten clinical cases were recalculated with the Encompass TPS model (Encompass_TPS_) and compared to the clinical plan, where the Encompass system was taken into account in the external contour (Encompass_EXT_). The targets in the ten clinical cases ranged in location as well as in size. The treatment techniques included volumetric modulated arc therapy (VMAT) and dynamic conformal arc (DCA); both techniques are implemented for SRS treatments at our institution. Table 1 summarizes the lesion location, size, and treatment technique for the 10 clinical cases recalculated.

**Table 1:** Summary of location, number of fractions, total dose, treatment technique and target volume for the 10 clinical cases in the study that were recalculated. Two plans treated two targets simultaneously, and the target volume for each target is shown. Percent difference in isocenter dose between measured and calculated with only Lucy (Lucy_only_) and with Lucy in the Encompass system (Lucy_ENC_ are summarized)

Location	Fractions	Total dose	Treatment technique	Target volume (cc)	Isocenter % difference, Lucy_only_	Isocenter % difference, Lucy_ENC_
Lt Acoustic Neuroma	1	14	VMAT	0.32	3.6%	4.7%
Lt Cerebellar + Lt Temporal^a^	1	18	VMAT	5.71, 7.96	−0.5%	−1.7%
Rt Parietal	1	18	DCA	0.16	−0.5%	0.2%
Rt Frontal	1	18	DCA	0.23	0.2%	1.1%
Rt Acoustic Neuroma	1	13	VMAT	6.75	0.5%	−0.2%
Lt Frontal	1	18	DCA	0.09	−2.1%	−0.2%
Lt Occipital	1	18	DCA	0.56	0.7%	1.1%
Rt Cavernous Sinus	3	24	VMAT	5.55	−1.3%	−2.0%
Lt Frontal	1	18	VMAT	14.0	1.6%	−0.3%
Lt Cerebellar^a^	1	18	VMAT	0.99, 0.04	2.1%	5.0%

a Two lesions treated simultaneously with a single isocenter, dose measured at the center of larger of the two lesions.

The dose to 99% (PTV_D99%_), 95% (PTV_D95%_), and 0.035 cc (PTV_D0.035cc_) of the PTV was evaluated between the clinical plan and the same plan recalculated with the Encompass TPS model. For the highest priority organ‐at‐risk, the dose to 0.035 cc (OAR_D0.035cc_) was evaluated. For the acoustic neuroma cases, the OAR with the highest priority is the cochlea. However, for a solitary metastasis with no physiological OARs within a 2 cm radius, a 0.5 cm ring around the PTV was created to simulate an OAR around the lesion. A paired student t‐test was used to evaluate the differences in PTV and OAR dose between the two plans, where *P* < 0.05 was the threshold for statistical significance.

The plans were measured in the Lucy_only_ setup and the Lucy in the Encompass insert using a PTW Pinpoint ion chamber (Freiburg, Germany), positioned at the isocenter. The measurements were performed at full couch rotations to include different portions of the Encompass mask system in the measurements. The difference between the Lucy_only_ and Lucy in the Encompass insert setup was compared to evaluate the overall attenuation through the Encompass insert.

### 2.F Couch placement sensitivity

The Encompass TPS model is manually registered and inserted on each patient image set. To evaluate the dosimetric uncertainty of the placement of the couch, the position of the couch model was intentionally displaced 3 mm in the vertical, lateral and longitudinal directions. This deviation was introduced to the Lucy phantom image set and the difference in dose to isocenter was evaluated between the intentional deviation and the baseline image set. The couch deviation was also applied to a patient image set, which showed the greatest discrepancy in dose calculation between the Encompass_TPS_ and Encompass_EXT_ images sets. For the patient data set, the dose to PTV_D99%_, PTV_D95%_, PTV_D0.035 cc_, OAR_D0.035 cc_, and point dose for each beam at isocenter were compared.

## 3 RESULTS

### 3.A Attenuation

In Zones 1–3, the average percent attenuation measured, Atten_Measured,_ for the 6xFFF beam was 3.8%, 3.6%, and 3.4%, for the 10xFFF beam 3.4%, 3.1%, and 2.9% and for the 6X beam 3.6%, 3.4%, and 3.3%, for field sizes of 2 × 2 cm^2^, 4 × 4 cm^2^, and 6 × 6 cm^2^, respectively. The largest amount of attenuation occurs in Zone 4, in the area where the mask attaches to the Encompass insert. The maximum attenuation measured in Zone 4 for the 6xFFF beam was 17.0%, 15.8%, and 15.2%, for the 10xFFF beam 12.7%, 12.3%, and 11.6% and for 6X beam 14.8%, 13.9%, and 13.7%, for field sizes of 2 × 2 cm^2^, 4 × 4 cm^2^, and 6 × 6 cm^2^, respectively. Table 2 summarizes the percent attenuation measured for the three energies and field sizes measured.

**Table 2:** Summary of the percent attenuation measured using a pinpoint ion chamber for 6X, 6xFFF, and 10xFFF photon energies for field sizes of 2 × 2 cm^2^, 4 × 4 cm^2^, and 6 × 6 cm^2^. The average percent attenuation (minimum, maximum) values are shown for Zones 1–3 and separately for Zone 4 where more attenuation is observed

Field Size	Location	6X	6xFFF	10xFFF
2 × 2 cm^2^	Zone 1–3	3.6% (2.9, 4.3%)	3.8% (3.2, 4.5%)	3.4% (2.8, 4.0%)
	Zone 4	8.0% (2.2, 14.8%)	8.9% (2.3, 17.0%)	7.2% (1.9, 12.7%)
4 × 4 cm^2^	Zone 1–3	3.4% (2.6, 4.2%)	3.6% (2.8, 4.4%)	3.1% (2.5, 3.6%)
	Zone 4	7.6% (2.1, 13.9%)	8.5% (1.8, 15.8%)	6.8% (2.1, 12.3%)
6 × 6 cm^2^	Zone 1–3	3.3% (2.5, 4.0%)	3.4% (2.6, 4.0%)	2.9% (2.4, 3.5%)
	Zone 4	7.3% (1.9, 13.7%)	8.2% (2.0, 15.2%)	6.5% (1.8, 11.6%)

The average difference between measured, Atten_Measured,_ and calculated attenuation, Atten_Calc,_ for the Encompass_EXT_ with the 6xFFF beam was −0.1%, 0.1%, 0.1%, for the 10xFFF beam was 0.3%, 0.3%, 0.4%, and for the 6X beam 0.2%, 0.3%, 0.4%, for field sizes of 2 × 2 cm^2^, 4 × 4 cm^2^, and 6 × 6 cm^2^, respectively. The maximum difference between measured and calculated attenuation with the Encompass_EXT_ was 3.6% which occurred in Zone4 for the 6xFFF beam for a 2 × 2 cm^2^ field size.

### 3.B HU validation

The HU values of the components of the Encompass systems are summarized in Table 3. The final HU value chosen for the Encompass_TPS_ was 400HU for the Encompass structure and −400 for the Encompass base structure. This minimized the difference between measured and calculated attenuation, (Atten_Calc_–Atten_Measured_). The percent difference between the measured and calculated attenuation for the Encompass_TPS_ for HU evaluation is summarized in Table 4.

**Table 3:** Summary of components of the QFix Encompass^TM^ SRS immobilization system, corresponding HU value ranges, and whether the component is included in the Encompass TPS model

	HU Range	Included in TPS Model
Insert (frame)	150 to 450	Yes (Encompass)
Insert (inner layer)	−900 to −950	Yes (Encompass Base)
Alignment Pins	50 to 85	No
Clips	35 to 95	No
Adjustable Shims	−450 to −50	No
Mask	30 to 150	No

**Table 4:** Evaluation of HU values for Encompass Insert and Base structures for the Encompass TPS model. The percent difference between measured and calculated attenuation is shown for 6xFFF, 10xFFF, and 6X for field sizes of 2 × 2 cm^2^, 4 × 4 cm^2^, and 6 × 6 cm^2^. HU values of 200, 300, 400 were evaluated for the frame structure and −600, −500, −400 for the base structure

	Encompass insert (HU)			Encompass base (HU)		
6xFFF	200	300	400	−600	−500	−400
2 × 2	0.53% ± 0.28%	0.38% ± 0.28%	0.24% ± 0.35%	0.73% ± 0.50%	0.46% ± 0.45%	−0.46% ± 0.45%
4 × 4	0.54% ± 0.41%	0.43% ± 0.40%	0.30% ± 0.44%	0.83% ± 0.51%	0.53% ± 0.44%	−0.08% ± 0.44%
6 × 6	0.65% ± 0.27%	0.53% ± 0.30%	0.38% ± 0.30%	0.88% ± 0.42%	0.61% ± 0.40%	0.01% ± 0.36%
10xFFF	200	300	400	−600	−500	−400
2 × 2	0.85% ± 0.24%	0.77% ± 0.24%	0.60% ± 0.29%	1.18% ± 0.41%	0.94% ± 0.40%	0.40% ± 0.41%
4 × 4	0.74% ± 0.19%	0.63% ± 0.19%	0.53% ± 0.23%	0.95% ± 0.39%	0.74% ± 0.19%	0.27% ± 0.37%
6 × 6	0.69% ± 0.37%	0.60% ± 0.41%	0.49% ± 0.41%	1.00% ± 0.33%	0.78% ± 0.30%	0.33% ± 0.29%
6X	200	300	400	−600	−500	−400
2 × 2	0.77% ± 0.28%	0.65% ± 0.33%	0.52% ± 0.33%	1.06% ± 0.40%	0.84% ± 0.33%	0.27% ± 0.43%
4 × 4	0.79% ± 0.40%	0.71% ± 0.41%	0.58% ± 0.43%	1.06% ± 0.35%	0.84% ± 0.30%	0.27% ± 0.33%
6 × 6	0.97% ± 0.36%	0.83% ± 0.39%	0.70% ± 0.39%	1.10% ± 0.41%	0.89% ± 0.34%	0.37% ± 0.34%

In Zones 1–3, the average difference between measured, Atten_Measured,_ and calculated attenuation, Atten_Calc,_ for the Encompass_TPS_ for the 6xFFF beam was −0.3%, −0.2%, and 0.0%, for the 10xFFF beam 0.2%, 0.2%, and 0.3% and for 6X beam 0.1%, 0.2%, and 0.4%, for field sizes of 2 × 2 cm^2^, 4 × 4 cm^2^, and 6 × 6 cm^2^, respectively. The maximum difference between measured and calculated attenuation with the Encompass_TPS_ was 3.0% which occurred in Zone4 for the 6X beam for a 6 × 6 cm^2^ field size. The percent differences are relatively similar in range to those obtained when dose was calculated from HU values obtained directly from the CT in the Encompass_EXT_ calculations. Figure 2(b) shows the measured and calculated percent attenuation from the Encompass_EXT_ and Encompass_TPS_.

### 3.C Clinical case recalculation and measurement

The average difference in PTV coverage between the patient data sets including the Encompass insert in the external with the TPS model for the ten patient plans for PTV_D99%_ was −0.04% and −0.07% for PTV_D95%_ for. The average difference to the maximum dose PTV_D0.035 cc_ was −0.08%. The average difference to the OAR_D0.035 cc_ was −0.2%. No significant difference, *P* > 0.05, was found between the two different image sets.

The absolute percent difference between the measurement with and without the insert ranged from −1.9% to 2.9% (Table 1). The average difference was 0.3% for all ten patients.

### 3.D Couch placement sensitivity

In Zones 1–3, the average percent difference in dose calculated on the Lucy when the Encompass insert TPS model was shifted 3 mm in the vertical, lateral, and longitudinal directions were 0.00%, −0.02%, −0.09%, respectively. The maximum difference observed was 0.45, 0.30%, and 0.0% in the vertical, lateral, and longitudinal directions, respectively. Whereas, the average difference in Zone 4 were 0.11%, 0.00%, and 0.12% when the TPS model for the Encompass insert was shifted 3 mm in the vertical, lateral, and longitudinal directions, respectively. The maximum difference was 3.19%, 0.34%, and 0.83% in the vertical, lateral, and longitudinal directions, respectively. The greatest percent difference in Zone 4 for all three translational directions was observed at Gantry 270°.

With a 3 mm translation displacement of the Encompass insert TPS model, the average difference for the clinical case recalculation for PTV_D99%_, PTV_D95%_, PTV_D0.035 cc_, and OAR_D0.035 cc_ was 0.02%, 0.02%, 0.003%, and −0.1%, respectively. The average percent difference at isocenter for the three treatment beams was 0.04%, 0.00%, and 0.07% for beam 1 at couch 0°, beam 2 at couch 280° and beam 3 at couch 40°, respectively. The maximum difference occurred at beam3, couch 40° when the couch was shifted 3 mm laterally.

## 4 DISCUSSION

A treatment planning system model of the QFix Encompass^TM^ SRS immobilization system was created as part of the HyperArc^TM^ High‐definition radiotherapy automated SRS delivery workflow. The TPS Encompass model is used to locate the patient in space relative to the treatment isocenter to ensure machine clearance during automated treatment delivery. If the Encompass insert is used independent of the HyperArc^TM^ workflow, it is not necessary to insert the couch model. However, to reduce dose calculation uncertainty due to areas of high attenuation, the Encompass insert should be included in the external contour. Areas of high attenuation, up to 17%, were observed in the region where the customized mask attaches to the Encompass insert which can impact the dose distribution if not considered in the dose calculation.

The magnitude of calculated attenuation between the Encompass_EXT_ and Encompass_TPS_ was similar. Differences in Zones 1 can be attributed to the way the Encompass base is modeled in the TPS model (Fig. 2). The base layer of the Encompass insert consists of a double layer of high‐density carbon fiber with a hollow center. The base layer was modeled as a solid piece due to the difficulty of modeling a thin layer of material in the TPS and transferring the structure to subsequent patient image sets. Due to this, the Encompass base layer HU value is effectively an average of the Encompass insert (400) and air (−1000). Due to an increase in equivalent path length when traversing a larger portion of the high density insert at an oblique angle, compared to the perpendicular entry through the base structure, the differences are accentuated when the base is modeled with only one material. The HU value of −400 was chosen for the Encompass base structure in order to minimize the average difference in Zone 1, which more closely match the attenuation through the oblique angles. If the attenuation was matched more closely at Gantry 180, the difference in attenuation at the oblique angles would increase. Differences in Zone 4 can be attributed to the difficulty and inherent inaccuracy in measuring and calculating dose for small fields in areas of heterogeneity.^12^, ^13^ Zone 4 includes portions of the insert, mask, adjustable shims and acrylic pins. The maximum difference in measured and calculated attenuation was within 5%, which is within the uncertainty between measured and calculated dose found in previous studies.^9^, ^13^, ^14^

**Figure 3:**
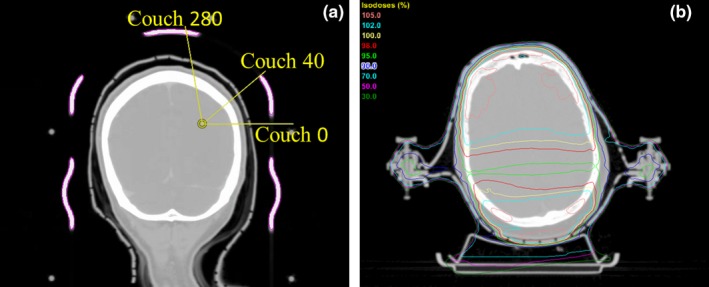
(a) Coronal cross‐section of clinical patient recalculated with translational shifts demonstrating sensitivity of positioning of beams relative to high density portions of the Encompass insert (magenta). (b) Axial cross‐section of two field, opposed lateral beams for whole brain radiotherapy treatment demonstrating areas of high attenuation through the clips resulting in decreased coverage to the brain.

It is important to include areas of high attenuation in the beam path to be included in the dose calculation. The TPS model is registered manually which may lead to potential errors in placement of the couch model. With a 3 mm translation shift in the vertical, longitudinal, and lateral directions, a maximum difference of 3% was observed for a static beam at gantry 270 through the clips and shimming system of the mask. The difference in couch shift was minimized in the actual treatment plan that consists of several non‐coplanar arcs. This minimizes the amount and regions of the higher density insert that the primary beam passes through. The sensitivity of the couch setup is very dependent on the location of the lesion, couch and gantry angles and what the treatment beam traverses. On the recalculated clinical plan, the percent change was within 1%; however, this can change depending on where the location of the lesion is relative to the high density portions of the insert [Fig. 3(a)]. Depending on the couch angle, the change in isocenter dose varies between beams. During the SRS planning process, arc geometry is typically chosen to achieve conformal dose distributions rather than to avoid portions of the mask that are more attenuating. However, for static fields, such as IMRT or 3D conformal techniques, care should be taken to avoid areas of high attenuation since the dosimetric consequences can be accentuated. Figure 3(b) demonstrates an example of whole brain opposed lateral radiotherapy treatment for a patient initially simulated for an SRS treatment. Areas of high attenuation occur at the clip area, resulting in decreased coverage to the brain. The patient was ultimately treated with whole brain using a hippocampal sparing VMAT technique.

In this study, the anisotropic analytical algorithm (AAA) was used to verify the HU values of the TPS model. For AAA, inaccuracies in dose calculation have been demonstrated at the interfaces of materials.^13^, ^14^ In the Encompass mask system, dose inaccuracies can occur between the mask, insert, and air. However beyond the interface region, dose from AAA is often comparable to Monte Carlo based algorithms, within 2%–4%.^15^ Other studies have also found that couch models included in the external body contour for dose calculation often agree with measurements within 2%.^5^, ^9^, ^11^

A limitation of this study is that skin dose was not evaluated. Because the mask is an integral part of the Encompass system and is customized for each patient, the mask acts as additional build up and the amount may vary from patient to patient. Also, for rotational type techniques such as DCA or VMAT, dose to the skin is often spread out across the arc path length. Furthermore, the largest dosimetric differences occurred at gantry 270 through the shimming system of the mask. The change in attenuation when the shimming level is adjusted was not evaluated, and may change as air gaps are introduced in the system. Future studies could be performed evaluating the dose to skin, as well as the impact of shimming level, static treatment fields as well as using more accurate algorithms such as Monte Carlo.

## 5 CONCLUSION

Significant attenuation occurs when using the QFix Encompass^TM^ SRS immobilization system, and occurs at the area where the mask attaches to the insert. HU values for the Encompass TPS model were found to be 400 for the Encompass structure and −400 for the Encompass base structure, which resulted in an average percent difference between measured and calculated attenuation of less than 0.5%. Small uncertainties in couch placement do not significantly perturb the dose calculation. However, larger differences can be seen when using few static beams compared to rotational treatment techniques.

## CONFLICTS OF INTEREST

This work was supported in part by a grant with Varian Medical Systems. Ning Wen, PhD was supported by a Research Scholar Grant, RSG‐15‐137‐‐01‐CCE from the American Cancer Society.

## References

[1] Ramakrishna N , Rosca F , Friesen S , et al. A clinical comparison of patient setup and intra‐fraction motion using frame‐based radiosurgery versus a frameless image‐guided radiosurgery system for intracranial lesions. Radiother Oncol. 2010;95:109–115. 20116123 10.1016/j.radonc.2009.12.030

[2] Verbakel WFAR , Lagerwaard FJ , Verduin AJE , Heukelom S , Slotman BJ , Cuijpers JP . The accuracy of frameless stereotactic intracranial radiosurgery. Radiother Oncol. 2010;97:390–394. 21047692 10.1016/j.radonc.2010.06.012

[3] Masi L , Casamassima F Polli C , Menichelli C , Bonucci I , Cavedon C . Cone Beam CT image guidance for intracranial stereotactic treatments: comparison with a frame guided set‐up. Int J Radiat Oncol Biol Phys. 2008;71:926–933. 18514784 10.1016/j.ijrobp.2008.03.006

[4] Breneman JC , Steinmetz R , Smith A , Lamba M , Warnick RE . Frameless image‐guided intracranial stereotactic radiosurgery: clinical outcomes for brain metastases. Int J Radiat Oncol Biol Phys. 2009;74:702–706. 19231101 10.1016/j.ijrobp.2008.11.015

[5] Smith DW , Christophides D , Dean C , Naisbit M , Mason J , Morgan A . Dosimetric characterization of the iBEAM evo carbon fiber couch for radiotherapy. Med Phys. 2010;37:3595–3606. 20831067 10.1118/1.3451114

[6] Olch AJ , Lavey RS . Reproducibility and treatment planning advantages of a carbon fiber relocatable head fixation system. Radiother Oncol. 2002;65:165–168. 12464445 10.1016/s0167-8140(02)00282-7

[7] Njeh CF , Raines TW , Saunders MW . Determination of the photon beam attenuation by the Brainlab imaging couch: angular and field size dependence. J Appl Clin Med Phys. 2009;10:2979. 19692980 10.1120/jacmp.v10i3.2979 PMC5720553

[8] Olch AJ , Gerig L , Li H , Mihaylov I , Morgan A . Dosimetric effects caused by couch tops and immobilization devices: report of AAPM Task Group 176. Med Phys. 2014;41:061501. 24877795 10.1118/1.4876299

[9] Vanetti E , Nicolini G , Clivio A , Fogliata A , Cozzi L . The impact of treatment couch modelling on RapidArc. Phys Med Biol. 2009;54:N157–N166. 19351984 10.1088/0031-9155/54/9/N03

[10] Myint WK , Niedbala M , Wilkins D , Gerig LH . Investigating treatment dose error due to beam attenuation by a carbon fiber tabletop. J Appl Clin Med Phys. 2006;7:21–27. 10.1120/jacmp.v7i3.2247 PMC5722426 17533341

[11] Mihaylov IB , Corry P , Yan Y , Ratanatharathorn V , Moros EG . Modeling of carbon fiber couch attenuation properties with a commercial treatment planning system. Med Phys. 2008;35:4982–4988. 19070232 10.1118/1.2982135

[12] Das IJ , Ding GX , Ahnesjo A . Small fields: nonequilibrium radiation dosimetry. Med Phys. 2008;35:206–215. 18293576 10.1118/1.2815356

[13] Arnfield MR , Siantar CH , Siebers J , Garmon P , Cox L , Mohan R . The impact of electron transport on the accuracy of computed dose. Med Phys. 2000;27:1266–1274. 10902555 10.1118/1.599004

[14] Han T , Mikell JK , Salehpour M , Mourtada F . Dosimetric comparison of Acuros XB deterministic radiation transport method with Monte Carlo and model‐based convolution methods in heterogeneous media. Med Phys. 2011;38:2651–2664. 21776802 10.1118/1.3582690 PMC3107831

[15] Bush K , Gagne IM , Zavgorodni S , Ansbacher W , Beckham W . Dosimetric validation of Acuros XB with Monte Carlo methods for photon dose calculations. Med Phys. 2011;38:2208–2221. 21626955 10.1118/1.3567146

